# Sequence determinants of the folding properties of box C/D kink-turns in RNA

**DOI:** 10.1261/rna.063453.117

**Published:** 2017-12

**Authors:** Saira Ashraf, Lin Huang, David M.J. Lilley

**Affiliations:** Cancer Research UK Nucleic Acid Structure Research Group, MSI/WTB Complex, The University of Dundee, Dundee DD1 5EH, United Kingdom

**Keywords:** RNA structure, snoRNP, k-turns, metal ions, L7Ae

## Abstract

Folding properties differ markedly between kink-turns (k-turns) that have different biological functions. While ribosomal and riboswitch k-turns generally fold into their kinked conformation on addition of metal ions, box C/D snoRNP k-turns remain completely unfolded under these conditions, although they fold on addition of L7Ae protein. Sequence elements have been systematically exchanged between a standard ribosomal k-turn (Kt-7) that folds on addition of metal ions, and a box C/D k-turn. Folding was studied using fluorescence resonance energy transfer and gel electrophoresis. Three sequence elements each contribute in an approximately additive manner to the different folding properties of Kt-7 and box C/D k-turns from archaea. Bioinformatic analysis indicates that k-turn sequences evolve sequences that suit their folding properties to their biological function. The majority of ribosomal and riboswitch k-turns have sequences allowing unassisted folding in response to the presence of metal ions. In contrast, box C/D k-turns have sequences that require the binding of proteins to drive folding into the kinked conformation, consistent with their role in the assembly of the box C/D snoRNP apparatus. The rules governing the influence of sequence on folding properties can be applied to other standard k-turns to predict their folding characteristics.

## INTRODUCTION

Kink turns (k-turns) are structural elements found in double-stranded RNA (Klein et al. 2001) that can adopt a tightly kinked geometry, mediating long-range tertiary contacts and frequently acting as protein binding sites. k-turns are widespread in functional RNA molecules, including the ribosome, the tri-snRNP spliceosomal complex, snoRNPs, and riboswitches (for review, see Huang and Lilley 2016). The conditions that lead to folding into the kinked conformation differ between different classes of k-turns, in a manner that is evidently related to the biological function of the RNA in which they occur.

**FIGURE 1.**
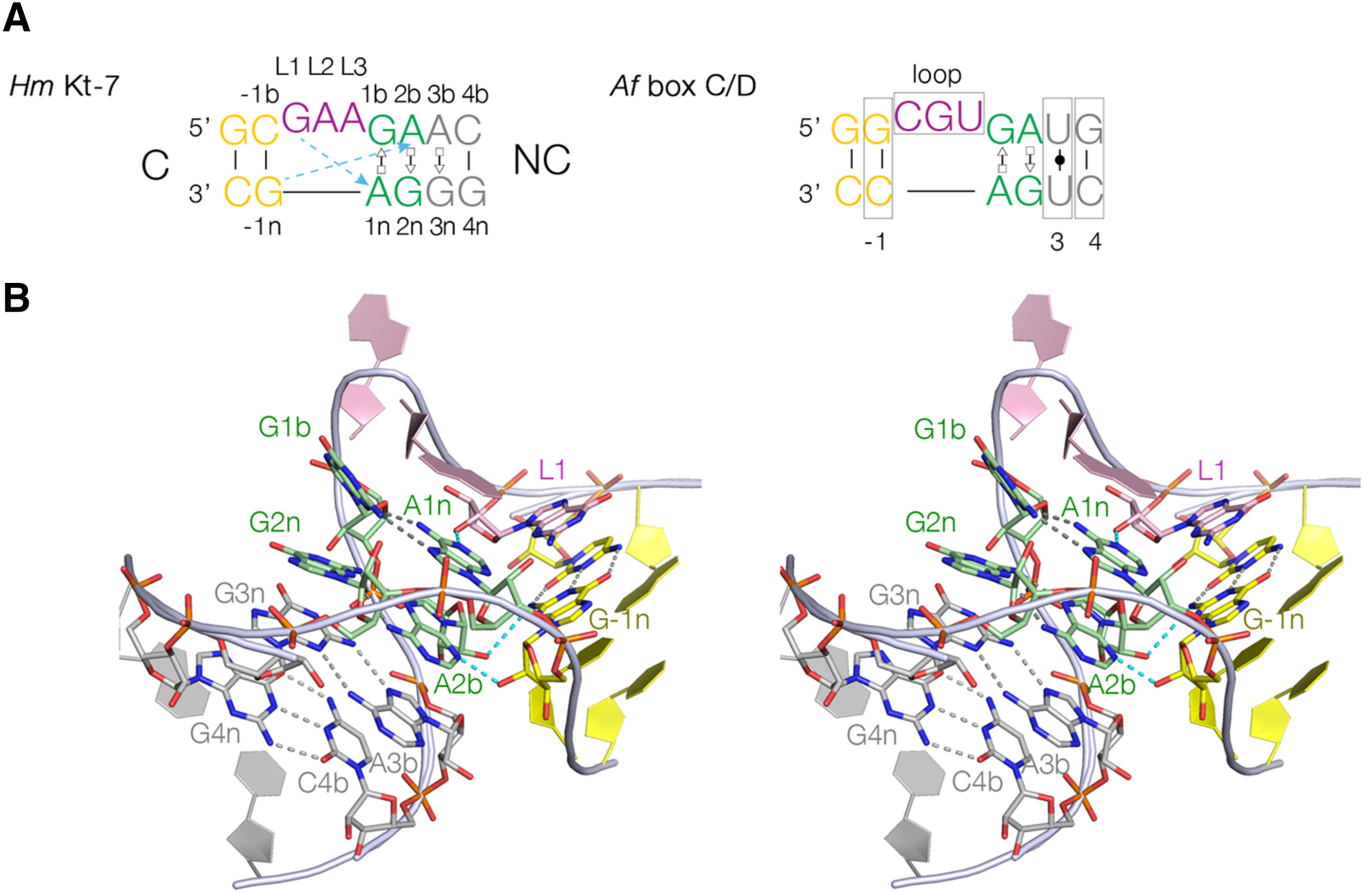
The k-turn structure in RNA. (*A*) The sequences of *H. marismortui* Kt-7 and *A. fulgidus* box C/D k-turns. The broken arrows colored in cyan indicate two critical cross-strand hydrogen bonds from the O2′ of L1 and −1n to the nucleobases of adenines 1n and 2b, respectively, that form the *trans* sugar–Hoogsteen G•A base pairs. The nomenclature of the nucleotide positions is shown on the Kt-7 sequence (*left*), and the various positions within the k-turn analyzed in this work are boxed on the box C/D sequence (*right*). In this view, the C-helix is on the *left*, while the NC-helix (that contains the G•A base pairs) is on the *right* side. (*B*) Parallel-eye stereoscopic image of the structure of the *H. marismortui* Kt-7 k-turn observed from the side of the non-bulged strand. The nucleotide positions that are studied and discussed in this work are identified. This image was prepared using PDB ID 4CS1, determined at 2.0 Å resolution (McPhee et al. 2014).

The standard k-turn comprises a 3-nucleotide (nt) bulge followed by tandem *trans* sugar–Hoogsteen G•A and A•G base pairs (Fig. 1). k-turns can fold into a kinked conformation, where the bulge is located at the apex and the axes include an angle of 50° (Daldrop and Lilley 2013). The nomenclature of the nucleotide positions in the k-turn (Liu and Lilley 2007) is indicated in Figure 1A. The k-turn folds by juxtaposing the minor grooves of the two helical arms, whereupon additional hydrogen bonds form across the interface. The nucleobases of the conserved adenine nucleotides of the G•A pairs accept hydrogen bonds from 2′-hydroxyl groups at the −1n and L1 positions in important, cross-strand interactions (Lescoute et al. 2005; Liu and Lilley 2007; Réblová et al. 2011; Daldrop and Lilley 2013).

Some k-turns fold into the kinked conformation on addition of metal ions (e.g., ∼100 µM Mg^2+^ ions), while some do not (McPhee et al. 2014). However, in general most k-turns fold upon binding specific proteins, especially the L7Ae class of RNA binding proteins (Turner et al. 2005; Wang et al. 2012; McPhee et al. 2014). For example, the ribosomal k-turn Kt-7 of *Haloarcula marismortui* adopts the kinked conformation in the presence of Mg^2+^ ions, whereas the human U4 snRNA k-turn remains unfolded in metal ions and requires the binding of the 15.5k protein (a member of the L7Ae family [Koonin et al. 1994; Nottrott et al. 1999]) to fold. We have previously shown that the identity of the base pair that follows the distal A•G pair (the 3b:3n base pair) determines the difference in folding characteristics between Kt-7 and the U4 k-turn (McPhee et al. 2014).

While the nature of the 3b:3n base pair correlated well with the folding characteristics of most k-turns (McPhee et al. 2014), there was one class that did not conform. This is the box C/D snoRNA that binds the L7Ae or 15.5k protein (in archaea and higher eukaryotes, respectively) as the first stage in the assembly of the box C/D snoRNP that directs the site-specific O2′-methylation of RNA (Kiss-László et al. 1996; Tycowski et al. 1996; Watkins et al. 2000, 2002; Tran et al. 2003; Bleichert et al. 2009; Ye et al. 2009; Xue et al. 2010; Lin et al. 2011). We have observed that the box C/D k-turn of *Archaeoglobus fulgidus* fails to undergo detectable folding in the presence of Mg^2+^ ions, studied either by fluorescence resonance energy transfer (FRET; this work) or by X-ray scattering inteferometry (Shi et al. 2016). This was a surprising result, because box C/D k-turn has 3b:3n = U:U, and when inserted into Kt-7 U:U at position 3b:3n results in a moderate degree of folding in metal ions (McPhee et al. 2014). Therefore, another element within the *A. fulgidus* box C/D k-turn sequence must influence the ion-dependent folding, completely preventing the folding of the box C/D k-turn.

Comparing the sequences of the *H. marismortui* Kt-7 and the *A. fulgidus* box C/D k-turns, given the conservation and role of the tandem G•A and A•G base pairs, the likely elements that might influence the folding properties are the −1b:−1n, loop, and 4b:4n sequence over and above the 3b:3n sequence already identified. We have therefore systematically exchanged these four sequence elements between Kt-7 and box C/D k-turns, and examined the ion-dependent folding of the hybrid k-turns by FRET. In this study we have only exchanged these elements, not explored the effect of a wider range of substitutions. Yet a clear result emerges from this study that in addition to the influence of the 3b:3n position, the −1b:−1n and 4b:4n sequences also affect the folding properties, of which the −1b:−1n position has a particularly strong influence. We show that the distribution of these sequences fit the biological requirements of the different k-turns, in particular differentiating those that are required to fold in metal ions alone from those that will require a bound protein to stabilize the folded geometry.

## RESULTS

### A comparative study of two k-turn sequences

In this work, we have made a systematic comparison of k-turn folding in the presence of Mg^2+^ ions by *H. marismortui* bacterial Kt-7 (*Hm* Kt-7) and the box C/D snoRNA from *A. fulgidus* (*Af* box C/D). These differ primarily in the −1b:−1n, loop, 3b:3n and 4b:4n sequences (Fig. 1). Unless it is explicitly stated otherwise, we shall simply refer to the two k-turns as Kt-7 and box C/D from here on. For these experiments, two series of molecules have been constructed in which the central k-turn is derived either from Kt-7 or box C/D. The sequences have been systematically varied, replacing the −1b:−1n, loop, 3b:3n or 4b:4n sequence with those from the other k-turn. The full sequences are presented in Materials and Methods; note that there are a few additional differences between the Kt-7 and box C/D outside the central k-turn region, and the C-helices are not identical in length, so that even when all four elements have been exchanged for a given species the resulting sequence is not identical to the other species.

### Systematic analysis of k-turn folding in Mg^2+^ ions for Kt-7 and box C/D as a function of sequence

**FIGURE 2.**
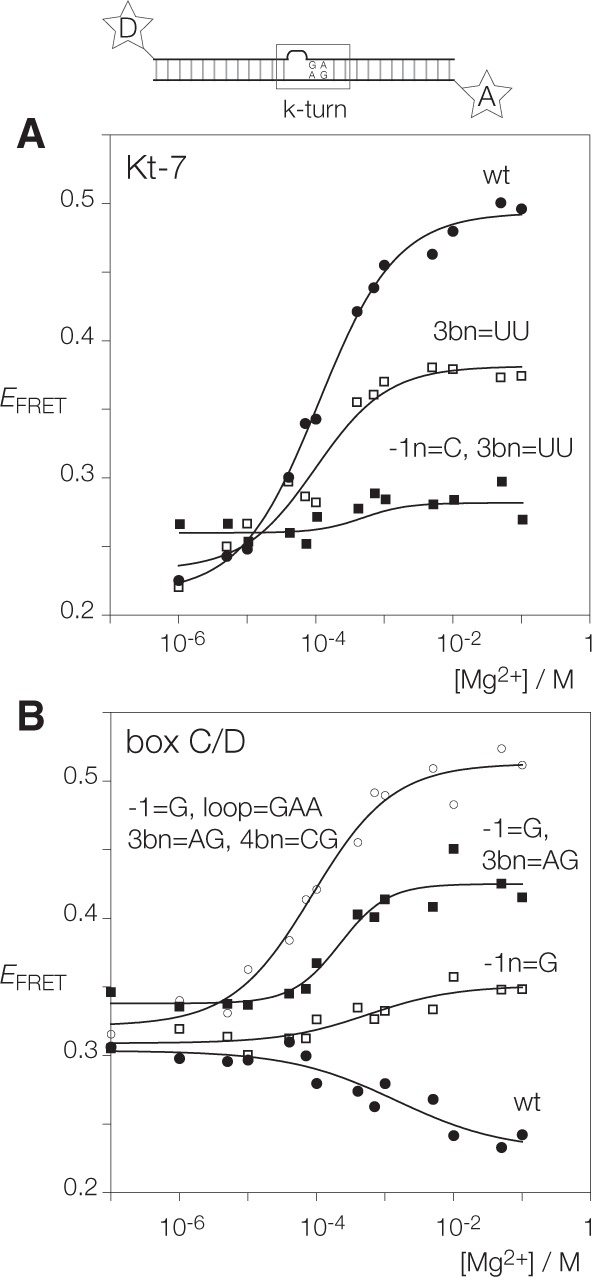
Analysis of ion-dependent folding of *H. marismortui* Kt-7 and *A. fulgidus* box C/D k-turns using FRET. The schematic (*top*) shows the constructs used in these studies. These are RNA duplexes with a central k-turn sequence, 5′-labeled with fluorescein donor (D) on the bulged strand and Cy-3 acceptor (A) on the nonbulged strand. The sequences are given in Materials and Methods. *E*_FRET_ is plotted as a function of Mg^2+^ ion concentration for both constructs (points), and fitted to the two-state model (line). (*A*) Titration of *Hm* Kt-7 unmodified from the natural sequence (wt, solid circles) and two representative variants in which sequence elements have been exchanged from the box C/D k-turn. The single-change variant in which 3b:3n = U:U (open squares) and the double-change variant with 3b:3n = UU plus −1b:−1n = G:C (filled squares) are shown. (*B*) Titration of *Af* box C/D unmodified from the natural sequence (wt, solid circles) and three representative variants in which sequence elements have been exchanged from the *Hm* Kt-7 k-turn. The single-change variant in which −1b:−1n = C:G (open squares), the double-change variant with −1b:−1n = C:G plus 3b:3n = A:G (filled squares), and the four-change variant with −1b:−1n = C:G, loop = GAA, 3b:3n = A:G and 4b:4n = C:G (open circles) are shown. Note that the four-change variant has effectively converted *Af* box C/D into *Hm* Kt-7. Data from titration of all *Hm* Kt-7 and *Af* box C/D k-turn variants are tabulated in Table 1.

We have analyzed k-turn folding in response to the addition of Mg^2+^ ions using a fluorescence resonance energy transfer (FRET) assay. We used k-turn-containing RNA duplex constructs labeled with fluorescein donor and Cy3 acceptor at the two 5′-termini (Fig. 2). Emission spectra were recorded in the steady state as a function of Mg^2+^ ion concentration, and the efficiency of energy transfer (*E*_FRET_) calculated using the acceptor normalization procedure (Clegg 1992). If the k-turn adopts the kinked conformation on addition of metal ions or protein binding, the end-to-end distance shortens, and therefore the *E*_FRET_ between the terminally attached fluorophores increases. These data have been fitted to a two-state folding model.

**FIGURE 3.**
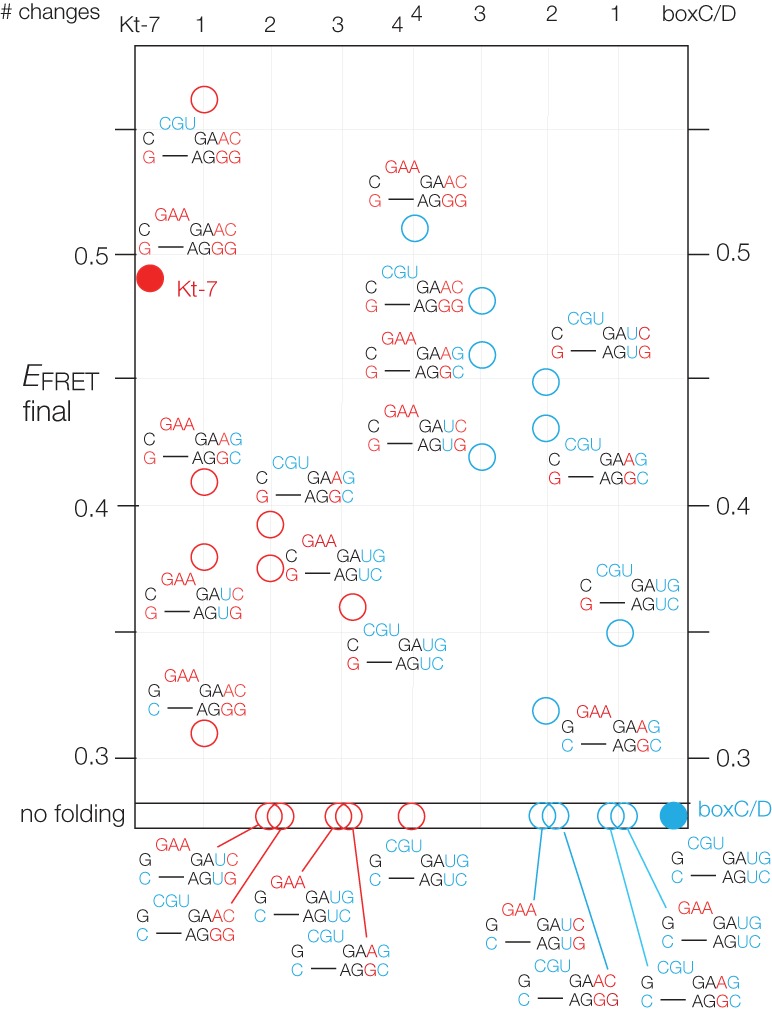
FRET analysis of ion-dependent folding of Kt-7 and box C/D k-turns and their variants with exchanged sequence elements. Final values of *E*_FRET_ in titrations with Mg^2+^ ions for *Hm* Kt-7 and *Af* box C/D k-turns and their variants. Species derived from *Hm* Kt-7 are shown in red, and those derived from *Af* box C/D are shown in blue. The unmodified species are shown with filled circles and all the variants with open circles. Those for which no folding was detected by FRET are drawn *below* the line at the base of the plot. All values of final *E*_FRET_ are given in Table 1.

Representative titrations are plotted in Figure 2, and the full set of initial and final values of *E*_FRET_ are tabulated in Table 1. In addition, final *E*_FRET_ values are plotted in Figure 3. It is readily apparent that the unmodified Kt-7 and box C/D sequences respond very differently upon addition of Mg^2+^ ions. For Kt-7, *E*_FRET_ increases over the range, consistent with folding into the k-turn conformation in the presence of the metal ions, and the data are well fitted by the two-state folding model giving a [Mg^2+^]_1/2_ = 110 µM. In contrast, for box C/D the initial value of *E*_FRET_ was higher, consistent with a more kinked conformation in the unfolded state, which then decreases on addition of Mg^2+^ ions. There is no detectable folding of the box C/D construct into the k-turn conformation in response to the addition of metal ions. However, addition of 1 µM L7Ae resulted in a final *E*_FRET_ of 0.63, corresponding to complete folding of the k-turn. Thus the box C/D k-turn is capable of adopting the k-turn conformation when bound by L7Ae, despite not being folded by metal ions alone.

**TABLE 1.**
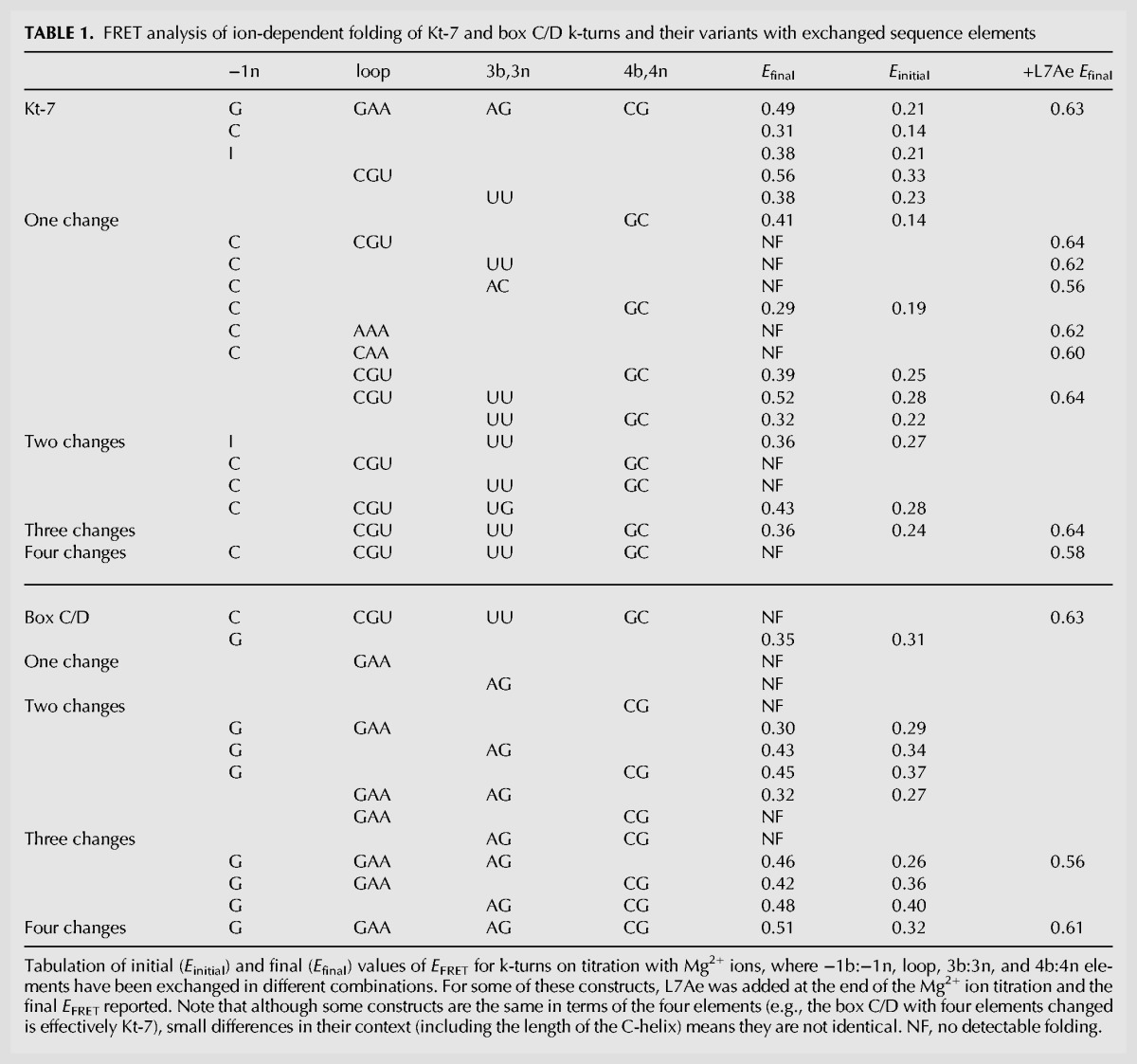
FRET analysis of ion-dependent folding of Kt-7 and box C/D k-turns and their variants with exchanged sequence elements

The titrations reveal changes of behavior for both species as the key sequence elements are exchanged. For Kt-7, all changes resulted in a reduced extent of k-turn folding, except when the loop was replaced by the box C/D loop CGU when we observed a small increase in the endpoint of *E*_FRET_. For box C/D, introduction of Kt-7 sequence elements tended to result in an increased extent of folding. We discuss the effect of these changes in detail below, but note here that the effects of sequence changes are approximately additive. In some cases we have added 1 µM L7Ae following the titration with Mg^2+^ ions. In all cases this results in an *E*_FRET_ of >0.55, irrespective of the extent of impairment of folding in Mg^2+^ ions.

### Effect of sequence changes on Kt-7 folding in Mg^2+^ ions analyzed by gel electrophoresis

**FIGURE 4.**
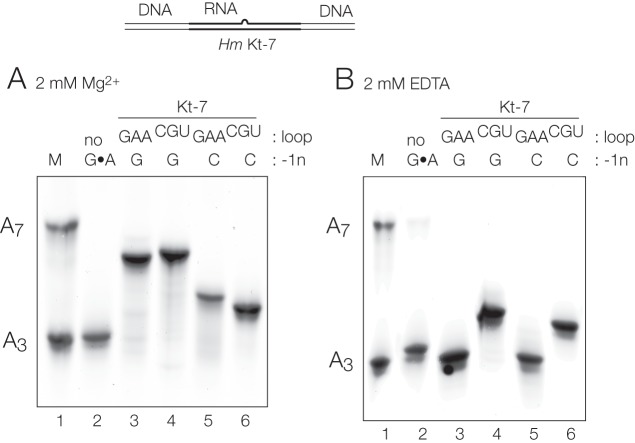
Analysis of ion-dependent folding of *H. marismortui* Kt-7 k-turn using gel electrophoresis. The schematic (*top*) shows the constructs used in these studies. The k-turn is centrally located in an RNA section embedded within a longer DNA segment giving a total length of 65 bp excluding the bulge. The different species were electrophoresed in a 13% polyacrylamide gel in the presence of (*A*) 2 mM Mg^2+^ ions or (*B*) 2 mM EDTA. To provide a frame of reference, equivalent duplexes in which the k-turn was replaced by an A_3_ or A_7_ bulge were mixed and electrophoresed in track 1. Track 2 contains a Kt-7 variant where the three G•A base pairs have been changed to Watson–Crick G–C base pairs by the substitutions of A1n, A2b, and A3b to C. Tracks 3 to 6 contain *Hm* Kt-7 variants with various substitutions taken from *Af* box C/D k-turn. Track 3, unmodified Kt-7; track 4 loop changed to CGU; track 5, −1b:−1n = G:C; track 4, −1b:–1n = G:C plus loop = CGU.

For a subset of the Kt-7 sequences, we have also used gel electrophoresis as an alternative means of studying folding in the presence of Mg^2+^ ions. These constructs comprised 65 bp DNA/RNA/DNA duplexes with a central core of RNA containing Kt-7 or a modified variant thereof. These were electrophoresed in a 13% polyacrylamide gel under nondenaturing conditions in recirculated buffer containing 2 mM MgCl_2_. To provide a reference, a mixture of two equivalent duplexes containing A_3_ and A_7_ bulges in place of k-turns was electrophoresed alongside the Kt-7 samples. The resulting gel (Fig. 4A) shows that the natural-sequence Kt-7 migrates alongside the A_7_ bulge, consistent with the tightly kinked k-turn conformation. However, when the k-turn is removed by converting all three A•G into C–G base pairs it then migrates with the A_3_ bulge, i.e., it has a similar degree of bending to a standard 3-nt bulge. Exchanging the −1b:−1n base pair with or without the loop to their equivalents from box C/D leads to significantly lower electrophoretic retardation, i.e., less kinking in the population, consistent with the FRET results.

### −1n is a key nucleotide determining the folding characteristics of Kt-7 compared to box C/D k-turns

The clearest result that emerges from this study is that the major determinant of the difference between the folding properties of Kt-7 and box C/D is the sequence at the −1b:−1n position. In *Hm* Kt-7 (and indeed in the great majority of k-turns) −1b:−1n is C:G, whereas in the box C/D k-turn it is reversed to G:C. The −1n nucleotide makes a key cross-strand hydrogen bond in the core of the k-turn (see below). Looking at the ensemble of the FRET titration results, for every construct in which −1n = G the final *E*_FRET_ > 0.35. And for every construct in which −1n = C, the final *E*_FRET_ < 0.32 and does not exhibit detectable folding in Mg^2+^ ions. Looking at the single changes (i.e., where just one element has been exchanged), the lowest final *E*_FRET_ for Kt-7 results from converting −1b:−1n to G:C, and for box C/D the only single change that leads to detectable folding is −1b:−1n changed to C:G. For double and triple changes, the biggest effect results from exchange of the −1b:−1n position.

### A smaller but generally additive effect of the 3b:3n and 4b:4n base pairs on k-turn folding

Bearing in mind that in these experiments we only exchange Kt-7 and box C/D elements, the effects of changes at the 3b:3n and 4b:4n positions are less marked. The effect of the single change in Kt-7 3b:3n from A:G to U:U is the next largest after the −1b:−1n position, and consistent with the identification of 3b:3n = U:U as a middle-folding sequence in our earlier systematic study of Kt-7 (McPhee et al. 2014). For double and triple changes, exchanging 3b:3n to U:U in Kt-7 results in a further reduced final *E*_FRET_, while changing 3b:3n to A:G in box C/D consistently increases *E*_FRET_ to a similar degree.

The effect of exchanging the 4b:4n sequence is similar in direction and magnitude to the changes at 3b:3n, and approximately additive with the other changes. In general, changing Kt-7 4b:4n from C:G to G:C leads to lower folding, while the reverse change in box C/D leads to increased folding.

### The sequence of the loop influences k-turn folding to a very small degree

The least important sequence difference between Kt-7 and box C/D for k-turn folding is the loop, which are GAA and CGU, respectively. Making the single change in Kt-7 leads to a small increase in final *E*_FRET_, consistent with a small increase in electrophoretic retardation for the same species (Fig. 4), and examples of both loop sequences are found for k-turns folding within the entire range of final *E*_FRET_ values. In general, exchange of loop sequences can lead to enhanced or impaired folding for different constructs, and the magnitudes are smaller than any of the other changes explored.

We note that the CGU loop sequence does lead to a higher value of *E*_FRET_ for the initial state prior to the addition of metal ions, suggesting that the unfolded k-turns (assumed to behave as simple three-nucleotide bulges) are on average more bent with that loop sequence. That is also supported by a retarded mobility of Kt-7 loop = CGU variant in gel electrophoresis in the absence of Mg^2+^ ions (Fig. 4B). We have previously noted that the magnitude kinking of base bulges depends on the sequence of the bulge (Bhattacharyya et al. 1990).

### Conservation of the −1n sequence in k-turns

**FIGURE 5.**
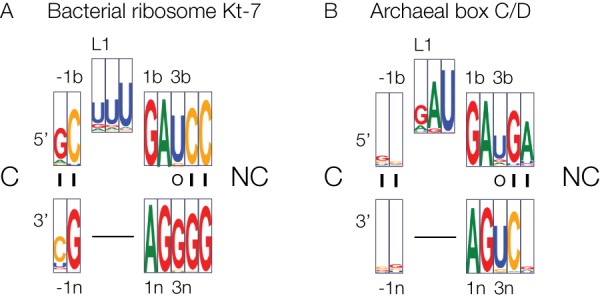
Bioinformatic analysis of nucleotide frequencies within bacterial ribosomal Kt-7 and archaeal box C/D k-turns. These data are presented in the form of WebLogo plots (Crooks et al. 2004) where the size of the letters is proportional to the frequency of that nucleotide at a given position. A similar analysis of the human box C/D k-turns made previously is similar to the archaeal plot shown here.

The single-most most important sequence difference determining the ion-dependent folding characteristics of Kt-7 and box C/D k-turns is the −1b:−1n base pair. In the majority of k-turns this is C:G, but in the *Af* box C/D k-turn it is reversed to G:C. We have used bioinformatics to analyze the distribution of k-turn sequences in the two k-turn types studied here. Nucleotide frequency distributions for bacterial Kt-7 and archaeal box C/D k-turns are shown in Figure 5 as Web-logo (Crooks et al. 2004) plots. Kt-7 exhibits a strong bias for −1b:−1n = C:G (Fig. 5A), predisposing the k-turns to ion-dependent folding. Moreover the 3b:3n base pair is most frequently U:G, which we have found to be well folded in Mg^2+^ ions (McPhee et al. 2014), and the 4b:4n base pair is C:G which we show here is also predisposed to ion-dependent folding. So each position in bacterial Kt-7 sequences has evidently evolved to facilitate folding in the presence of Mg^2+^ ions. More generally, the distribution frequency for −1b:−1n has been analyzed for the all the bacterial ribosomal k-turns by Westhof and coworkers (Lescoute et al. 2005), showing C:G = 94.3%; G:C = 3.0%; U:A = 1.7%; U:G = 0.8%; and A:U = 0.2%. Thus the great majority of sequences should be strongly predisposed to folding in the presence of divalent ions.

The nucleotide frequency distributions for archaeal box C/D k-turns (Fig. 5B) contrasts significantly with the ribosomal k-turns. The frequencies for the −1b:−1n sequences are C:G = 39.42%; G:C = 23.08%; A:U = 11.54%; U:U = 7.21%; G:U = 4.33%; and U:G = 2.89%. These are more widely distributed, and the second-most frequent −1b:−1n sequence is the non-ion-folding G:C. Moreover 3b:3n has a strong propensity to U:U, and 4b:4n is strongly conserved as G:C. Evidently box C/D sequences have evolved to be unfolded in divalent metal ions, and in general will require protein binding to fold into their kinked conformation.

### A role for the exocyclic amine of G-1n in k-turn folding

**FIGURE 6.**
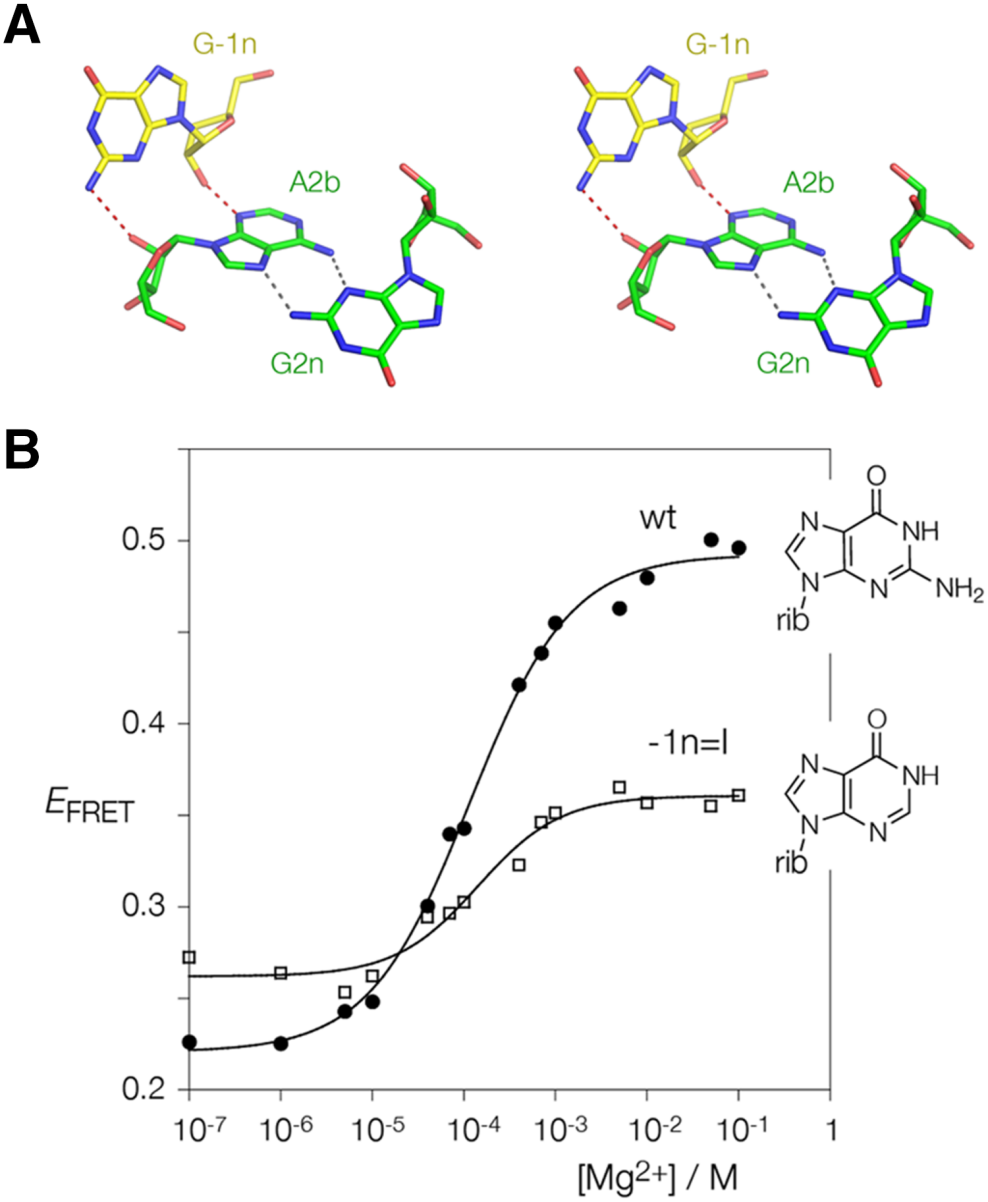
Atomic mutagenesis to probe the importance of the hydrogen bonding between G-1n and A2b. (*A*) Parallel-eye stereoscopic image of the interaction of G-1n with A2b in *Hm* Kt-7. The cross-strand hydrogen bonds are colored red. Note the additional hydrogen bond from G-1n N2 to the O2′ of A2b. This image was prepared using PDB ID 4CS1. (*B*) A modified version of the Kt-7 construct used for FRET analysis was prepared in which G-1n was replaced by inosine, thus replacing the amine at the 2 position with a proton. Folding as a function of Mg^2+^ ion concentration was studied by measurement of FRET efficiency between the terminally attached fluorophores as before. The data are plotted for unmodified Kt-7 (filled circles) and the G-1nI variant, and fitted to the two-state folding model. Note that the folding of the variant (open squares) lacking the G-1n N2 is significantly impaired.

We have established that the great majority of k-turns have −1b:−1n = C:G, and that if this base pair is reversed then folding in the presence of Mg^2+^ ions is severely impaired. The nucleotide at −1n makes one of the key cross-strand A-minor interactions in the core of the k-turn (Fig. 6A). The O2′ donates its proton to the nucleobase of A2b. The acceptor can be either N3 or N1, but in *Hm*Kt-7 (except within the ribosome) the proton is accepted by N3 (Daldrop and Lilley 2013). Additionally however, when −1n = G, the nucleobase can form a hydrogen bond from N2 to the O2′ of A2b. We therefore used atomic mutagenesis to assess the importance of this interaction to the Mg^2+^ ion-dependent folding of Kt-7. We selectively removed the exocyclic amine of A2b by inosine substitution and studied the folding in response to addition of Mg^2+^ ions using FRET as before (Fig. 6B). Folding is significantly impaired by removal of the N2 amine, achieving a final *E*_FRET_ of 0.36. Thus when −1b:−1n = C:G an additional cross-strand hydrogen bond forms, and this may help to tip the free energetic balance towards folding in the presence of Mg^2+^ ions.

## DISCUSSION

This study has demonstrated that the ribosomal k-turn *Hm* Kt-7 and the *Af* box C/D snoRNP k-turn have evolved sequences that dictate very different folding properties. By selectively exchanging segments between the two we have found that a number of sequence elements in the standard k-turn determine the folding properties in the presence of divalent metal ions that are suited to their function. These are principally the −1b:−1n, 3b:3n, and 4b:4n base pairs. We had previously established the importance of the 3b:3n base pair in determining whether or not a k-turn would fold in response to the presence of metal ions (McPhee et al. 2014), as well as the conformation adopted (Huang et al. 2016). We now find that additional elements can exert a significant influence on the folding properties in an approximately additive manner, particularly the −1b:−1n base pair. This generates a set of “rules” relating k-turn sequence to their ability to fold in the presence of Mg^2+^ ions. Those that cannot do so will require additional assistance to fold into the kinked conformation, most notably the binding of proteins such as members of the L7Ae family. We have observed that each k-turn whose sequence prevents ion-dependent folding will nevertheless fold in response to the binding of L7Ae, including the *Af* box C/D k-turn. Similarly, we have previously shown that human box C/D k-turns fold on binding the mammalian L7Ae homolog 15.5k protein (Huang et al. 2017).

Every element of the sequence of *Hm* Kt-7 predisposes it to fold in response to the presence of Mg^2+^ ions, with G at the critical −1n and 3n positions and 4b:4n = C:G. These elements are strongly conserved in all the bacterial Kt-7 sequences, and more generally in ribosomal k-turns. Most of these do bind various proteins within the ribosome, yet clearly they are not required in order to fold the k-turns. More likely the proteins bind after the folded state has been achieved to fix the structure of the assembling ribosome.

In marked contrast, the *Af* box C/D k-turn has evidently evolved to prevent folding in the absence of protein binding. 3b:3n = U:U is one of the intermediate ion-induced-folding k-turns (McPhee et al. 2014), and this is coupled with −1b:−1n = 4b:4n = G:C, both of which impair folding in metal ions. Many archaeal box C/D k-turns conform to this sequence pattern. Even where −1b:−1n = C:G, the combination of 3b:3n = U:U and 4b:4n = G:C results in very impaired ion-induced folding (Table 1). In human box C/D sequences, while the −1b:−1n is quite variable, 3b:3n = U:U and 4b:4n = G:C are strongly conserved (Huang et al. 2017). In the assembly of the box C/D snoRNP the first event is the binding of L7Ae (15.5k in humans), thereby generating the kinked conformation of the k-turn. This is then followed by the sequential binding of Nop5 (Nop56 plus Nop58 in humans) and finally fibrillarin, the methyl transferase enzyme (Watkins et al. 1998, 2002; Omer et al. 2002; Schultz et al. 2006; McKeegan et al. 2007). However, if the binding of L7Ae/15.5k is prevented, progression to further assembly is blocked (Watkins et al. 2002). Thus assembly of the box C/D snoRNP can in principle be controlled at the stage of the binding of L7Ae/15.5k to the k-turn. We have recently shown that in a subset of human box C/D sites this can be regulated by *N*^6^-methylation of A1n, which disrupts the G•A *trans* sugar–Hoogsteen base pair required to form the k-turn (Huang et al. 2017). However, methylation of the A1n requires that the nucleotide at −1n be cytosine, to create a GAC target for the METTL3-METTL14 methyl transferase (Dominissini et al. 2012; Meyer et al. 2012) and thus for these box C/D species −1b:−1n = G:C.

The occurrence of −1b:−1n = G:C in the box C/D appears to be largely restricted to this class of k-turn. Indeed, that was the reason the role of this position was not explored earlier. We have previously noted that 98% of U4 snoRNA k-turns have selected 3b:3n sequences that are incompatible with ion-induced folding, yet have −1b:−1n = C:G. Like the box C/D k-turns, these will require the binding of 15.5k protein in the pre-spliceosome to adopt the kinked geometry, but in general have adopted a different sequence strategy to achieve this.

We may ask how the sequence “rules” deduced from the Kt-7 and box C/D sequence exchange experiments apply to another class of k-turns, the riboswitches. Seven different riboswitch types employ k-turns as structural elements. For example, in the SAM-I riboswitch a long helix is kinked by a k-turn so that its terminal loop can make a tertiary interaction to generate the ligand binding site (Montange and Batey 2006). Bioinformatic analysis has shown that in 82% of the SAM-I k-turns the 3b:3n sequences confer ion-induced folding (McPhee et al. 2014), and this is reinforced by the −1b:−1n position where 97.7% have −1b:–1n = C:G (4757 sequences analyzed). Clearly the SAM-I riboswitch k-turns have a very strong requirement to undergo folding in the presence of metal ions alone, consistent with the absence of known proteins that bind these riboswitches.

In summary, a number of sequence elements in the k-turn contribute to its folding properties, and individual k-turns are adapted to the specific biological requirement. Those that are required to fold in response to metal ions alone will have different sequence elements from those that contribute to the biogenesis of ribonucleoprotein assemblies.

## MATERIALS AND METHODS

### RNA synthesis and deprotection

RNA oligonucleotides were synthesized by solid-phase phosphoramidite chemistry implemented on an ABI-394. The phosphoramidite ribonucleosides, Pac-A-CE, Ac-C-CE, iPr-Pac-G-CE, and U-CE, with a *t*-BDMS protecting group on the 2′O, were obtained from Link Technologies. The I-CE *t*-BDMS phosphoramidite was obtained from Glen Research. Fluorescein (Link Technologies) and Cy3 (GE Healthcare) were attached to the 5′ termini of the oligonucleotides as phosphoramidites in the final cycle of the synthesis, as required. For comparative gel electrophoretic experiments, DNA–RNA–DNA species were synthesized by incorporating deoxyribonucleosides. dT and Tac-protected dA, dG, and dC were obtained from Sigma-Aldrich.

The oligonucleotides were deprotected in 25% ethanol/ammonia solution for 3 h at 20°C. Fluorophore-labeled oligonucleotides, as well as DNA–RNA–DNA chimeras, were further deprotected for 2 h at 65°C. After deprotection, the oligonucleotides were evaporated to dryness. They were redissolved in 115 µL DMSO (Sigma-Aldrich) to which was added 60 µL triethylamine (TEA) (Sigma-Aldrich) and 75 µL 1 M triethylamine trihydrofluoride (TEA, 3HF) (Sigma-Aldrich) and incubated at 65°C for 2.5 h to remove the *t*-BDMS protecting groups. Thereafter, the samples were cooled on ice for 10 min and 250 µL RNA Quenching Buffer (Glen Research) was added. The oligonucleotides were then desalted by application to NAP-10 columns (GE Healthcare).

### RNA purification and hybridization

The oligonucleotides were purified by gel electrophoresis under denaturing conditions, using 20% acrylamide: bis-acrylamide (19:1) (Scientific Laboratory Supplies) electrophoresed in 90 mM Tris.borate (pH 8.5), 10 mM EDTA (TBE buffer) containing 7 M urea. Electrophoresis was performed at 25 W for ∼ 3 h. Nucleic acids were visualized by UV shadowing and bands corresponding to full-length products were excised and electroeluted into 140 µL 8 M ammonium acetate in TBE at 150 V at 20°C. The nucleic acid was then precipitated with ethanol.

Fluorophore-labeled oligonucleotides were subjected to further purification by reversed-phase HPLC on a C18 column (ACE 10-300, Advanced Chromatography Technologies), using an acetonitrile gradient with an aqueous phase of 100 mM triethylammonium acetate (pH 7.0) (Fisher Scientific). The samples collected were evaporated to dryness and resuspended in 120 µL ultrapure water.

For FRET experiments, equimolar quantities of the appropriate oligonucleotides were annealed in TBE, 25 mM NaCl, and slowly cooled from 95°C to 4°C. Hybridized RNA was purified by gel electrophoresis under nondenaturing conditions in 12% acrylamide: bis-acrylamide (29:1), in TBE, 25 mM NaCl with buffer recirculation. Electrophoresis was performed at 150 V at 4°C for ∼6 h. Bands containing duplex RNA were excised from the gel and electroeluted into 140 µL 8 M ammonium acetate in TBE at 100 V at 4°C, followed by ethanol precipitation and air-drying at 4°C.

### FRET analysis of k-turn folding

FRET efficiency was measured from a series RNA duplex species, terminally 5′-labeled with fluorescein on the bulged strand and Cy3 on the nonbulged strand, containing central k-turn sequences and variants (Fig. 2). The unmodified strands were (all sequences written 5′–3′):

*Kt-7*: Flu-CCAGUCAGUGGCGAAGAACCAUGUCAGG and Cy3-CCUGACAUGGGGAGCCACUGACUGG
*Box C/D*: Flu-CCUCAGUGGGCGUGAUGCAUGUCAUG and Cy3-CAUGACAUGCUGACCCACUGAGG

Hybridized together these generate C helices of 12 (Kt-7) and 10 (box C/D) bp, and NC helices of 13 bp (both constructs). Modified versions of these species were constructed as indicated in the text.

Absorption spectra were measured by resuspending the ethanol precipitated hybrid RNA in 120 µL 90 mM Tris-borate (pH 8.4) and recording the absorbance in a 50 mm path length cuvette using a NanoDrop 2000c spectrophotometer (Thermo Scientific). Spectra were deconvoluted using a corresponding RNA species labeled only with Cy3, and fluorophore absorption ratios calculated using a MATLAB program. Fluorescence spectra were recorded in 90 mM Tris-borate (pH 8.4) at 4°C using an SLM-Aminco 8100 fluorimeter. Spectra were corrected for lamp fluctuations and instrumental variations, and polarization artifacts were avoided by setting excitation and emission polarizers crossed at 54.7°. Values of FRET efficiency were measured using the acceptor normalization method (Clegg 1992) implemented in MATLAB. *E*_FRET_ as a function of Mg^2+^ ion concentration was analyzed on the basis of a model in which the fraction of folded molecules corresponds to a simple two-state process for ion-induced folding, i.e.,

$$E_{\mathrm{FRET}}=E_{0}+\Delta E_{\mathrm{FRET}}K_{A}{\left[{\text{Mg}}^{2+}\right]}^{n}/(1+K_{A}{\left[{\text{Mg}}^{2+}\right]}^{n}),$$

where *E*_0_ is the FRET efficiency of the RNA in the absence of added metal ions, Δ*E*_FRET_ is the increase in FRET efficiency at saturating metal ion concentration, [Mg^2+^] is the prevailing Mg^2+^ ion concentration, *K*_*A*_ is the apparent association constant for metal ion binding, and *n* is a Hill coefficient. Data were fitted to this equation by nonlinear regression. The metal ion concentration at which the transition is half complete is given by [Mg^2+^]_1/2_ = (1/*K*_*A*_)^1/^^*n*^.

### Gel electrophoretic analysis of k-turn folding

DNA–RNA–DNA duplex species were prepared by annealing equimolar quantities of the appropriate oligonucleotides in 50 mM Tris–HCl (pH 8.1), and slow cooling from 95°C to 4°C. The hybridized species were then electrophoresed in 13% acrylamide: bis-acrylamide (29:1) gels in 90 mM Tris-borate (pH 8.3) (TB buffer), 2 mM MgCl_2_ with buffer recirculation at >1 L h^−1^. Electrophoresis was performed at 120 V at 4°C for ≥72 h. Gels were stained using SYBR Gold (Life Technologies), washed in MilliQ water, and visualized on a Typhoon FLA 9500 (GE Healthcare).

The sequences used for the electrophoretic experiments are shown below.

Kt-7 upper strand:

CGCAAGCGACAGGAACCTCGCCAGUCAGUGGCGAAGAA CCAUGUCAGGGGACTGTCAAGTTGAACAGG

Kt-7 lower strand:

CCTGTTCAACTTGACAGTCCCCUGACAUGGGGAGCCAC UGACUGGCGAGGTTCCTGTCGCTTGCG

The DNA sections of these oligonucleotides are shown underlined. Nucleotide substitutions were introduced into the RNA sections as indicated in the text.

### Bioinformatic analysis

Archaeal box C/D sequences were taken from the box C/D snoRNA Database (http://lowelab.ucsc.edu/snoRNAdb/Annotations-archaea.html) (Omer et al. 2000). The k-turn region was aligned manually based on the known pattern of conserved nucleotides. A WebLogo plot (Crooks et al. 2004) showing the occurrence of box C/D sequences in archaea snoRNA was made using the website http://weblogo.threeplusone.com.

Bacterial Kt-7 sequences were taken from the Comparative RNA website (Cannone et al. 2002). Specific k-turn regions were aligned manually using Jalview 2.9 (Waterhouse et al. 2009). This resulted in the analysis 2812 of Kt-7 sequences.

SAM-I riboswitch sequences were taken from the Rfam database (Burge et al. 2013).

All sequence composition and covariation analysis was calculated using a modified version of Jalview 2.9 (Waterhouse et al. 2009), that was kindly provided by Dr. James Procter (University of Dundee).
